# Dr. Lorenzo S. Bartholemew

**Published:** 1906-02

**Authors:** 


DR. LORENZO S. BARTHOLEMEW, of Reading Center, N. Y., died at the Buffalo General Hospital, January 31, 1906, aged 49 years. He graduated from the Medical Department of the University of Buffalo in 1884. His illness was due to a disease of the spine for which a surgical operation was made a few days before his death.

